# Origin of Homochirality in Biosystems

**DOI:** 10.3390/ijms10031290

**Published:** 2009-03-18

**Authors:** Søren Toxvaerd

**Affiliations:** DNRF center “Glass and Time”, Roskilde University, Postbox 260 DK-4000 Roskilde, Denmark; E-Mail: st@ruc.dk

**Keywords:** Origin of chirality, Origin of Life, Prebiotic self-assambly

## Abstract

Experimental data for a series of central and simple molecules in biosystems show that some amino acids and a simple sugar molecule have a chiral discrimination in favor of homochirality. Models for segregation of racemic mixtures of chiral amphiphiles and lipophiles in aqueous solutions show that the amphiphiles with an active isomerization kinetics can perform a spontaneous break of symmetry during the segregation and self-assembly to homochiral matter. Based on this observation it is argued that biomolecules with a sufficiently strong chiral discrimination could be the origin of homochirality in biological systems.

## 1. Introduction

The homochirality of amino acids and sugar molecules in biosystems is a necessity for life, and the preservation of homochirality over long periods of time in a prebiotic fluid environment is *the* problem. It is *the* problem because it is not sufficient to obtain homochirality. Due to the active isomerization kinetics in fluid systems [1, 2], which in general drives a chiral system toward a racemic composition, it is of utmost importance to determine the condition and mechanism for preservation of homochirality in prebiotic systems. From a thermodynamical and physico-chemical point of view biosystems consist of big molecules of homochiral units and in a soft condensed state in an aqueous solution. If the origin of life is not based on an extremely rare event we therefore need an explanation of how to maintain homochirality in such a prebiotic state for millions of years. If the creation of ”simple” biosystems is obtained by a steady state synthesis, the prebiotic fluid state must necessarily have been rather constant over very long times in order to build up the very unique and complicated templates for life, where RNA is probable the original template. Even in a deterministically driven self-organisation such template-molecule is not established “overnight”. This is why a mechanism ensuring the preservation of homochirality for a very long time is needed. A period of time which is much longer than the time in which an (diluted) aqueous solution of an amino acid will racemize [1], i.e. in the order of thousands of years. Sugar molecules are much less stable than amino acids and racemize within hours or days depending on the physico-chemical conditions [2]; however, both key-molecules are central in all biosystems. Almost all theories dealing with homochirality have concentrated on explaining its origin, but, irrespective of whether L-amino acids float to us from Space or are obtained by a kind of autocatalysis, it is irrelevant, as we need to ensure its stability during long periods of time within the fluid state when biosystems were synthesized probably around 4 billion years ago. Finally it shall be emphasized that it is not sufficient to explain the origin and preservation of one of the homociral species in biosystems, e.g. D-sugars. One needs to explain the preservation of D-sugars as well as L-amino acids in a prebiotic fluid state.

The strong chiral discrimination of some of the central biomolecules offers such an explanation not only for a spontaneously break of symmetry and the origin of chirality [4], but also for the preservation of the homochiral states of D-sugar and L-amino acids.

### 2. Chiral Discrimination

The existence of chiral discrimination was discovered by Louis Pasteur in 1848 [3]. It manifests itself in the crystallization from a racemic fluid mixture of enantiomers into crystals of homochiral molecules, as demonstrated by Pasteur. As is well known, it is due to the fact that for some molecules there is an energy gain by packing homochiral molecules together instead of in pairs of enantiomers. The energy gain must, at least, be bigger than the temperature times the (ideal) mixing entropy for a mixing of equal amount of D- and L- forms, which is of the order *RTln*(2) ≈ 2 kJ/mole at room temperature. A measure of the strength of the chiral discrimination, Δ_cd_*H*^⊖^, can be given as the difference in (standard) formation enthalpies of the two compositions:

(1) $$\Delta_{\text{cd}}H^{⊖}=\Delta_{\text{f}}H^{⊖}(enantiomer)\;-\;\Delta_{\text{f}}H^{⊖}(racemic)$$

From a physico-chemical point of view biosystems are in a fluid state. Because the polymerization of amino acids, as well as of sugar and RNA, are dehydrations with a release of one water molecule per covalent polymerization bond, the state of synthesis of prebiotic systems is, what in physical chemistry is refered to by (aq) for the aqueous solution. On the other hand, the concentration of the chiral molecules at prebiotic self-assembling must necessarily have been high, with only a low concentration of water molecules, for the simple reason that the isomerization kinetics in a diluted aqueous solution drives the system toward a racemic composition. Therefore a measure of the strength of the chiral discrimination should be obtained for the fluid state (*l*): Δ_cd_*H*^⊖^ (*l*); however, this strength should at least be bigger than the (ideal) mixing entropy plus the chiral enthalpy loss and entropy gain caused by the presence of water molecules.

In general, physico-chemical data for the strength of chiral discrimination, Δ_cd_*H*^⊖^ (*l*) in the fluid state do not exist. Only for the simple triose glyceraldehyde (Gla) where Δ_cd_*H*^⊖^ (*l*)= −11.7 kJ/mole [5], which is much more than what is needed to overcome the mixing entropy. For some amino acids one can determine Δ_cd_*H*^⊖^ (*c*) for the enthalpy difference between an enantiomer crystal and a racemic crystal. This strength is, however, weakened at melting due to less effective packing in the fluid state. A collection of available data is given in Table 1. As can be seen from this table a few central amino acids might have the potential to maintain a homochiral liquid state, (l) or aqueous state (aq). The simple triose, glyceraldehyde (Gla) has, however, an exceptionally and sufficiently strong chiral discrimination.

## 3. The Isomerization Kinetics

The isomerization kinetics of amino acids and sugar are complex [1, 2, 6]. We shall simplify the reaction mechanism as a bimolecular reaction

(2) $$\text{D}+\text{D}\overset{E_{\text{DD}}}{\underset{E_{\text{DL}}}{⇌}}\;\text{D}+\text{L}\;\overset{E_{\text{DL}}}{\underset{E_{\text{LL}}}{⇌}}\;\text{L}+\text{L}$$

between the two chiral species. The activation energy, *E*_DL_, for DL-collisions, which may convert a D-molecule into a L-molecule or *vice versa*, is, in a condensed racemic fluid, less than the corresponding activation energy, *E*_DD_ = *E*_LL_, thus allowing a conversion of one of the molecules in the collisions. The inequality

(3) $$E_{\text{DL}}<E_{\text{DD}}=E_{\text{LL}}$$

accounts for the chiral discrimination with a lower potential energy of pairs in homochiral domains of one of the enantiomers corresponding to the effective strength of the chiral discrimination [7]. More specifically

(4) $$E_{\text{DL}}+\left|\Delta_{\text{cd}}H(x)\right|=E_{\text{DD}}=E_{\text{LL}},$$

where | Δ_cd_*H*(*x*) |, is a function of the *local* composition, *x*(r) of the molecules at the position r, and with a maximum (negative) value for a homochiral local composition.

A racemic D,L-system tends to separate in homochiral domains of enantiomers if the enthalpy gain, due to the chiral discrimination, is bigger than the entropy of mixing, just as in the case of Pasteur’s experiment, but now in a fluid state. Without isomerization kinetics such a racemic mixture will separate through molecular diffusion. An active isomerization kinetics enhances the separation; but what is much more significant, the kinetics also ensures a break of symmetry and results in the dominance of one of the species at late times [4].

The break of symmetry is obtained by, what could be expressed as *selfstabilizing chance* [8], whereby a local spontaneous concentration of a chiral species enhances the growth of the local domain due to the isomerization kinetics, which is active in the interface, whereas the chiral discrimination inside the homochiral domain slows down the conversion accordingly to Eq. (4). The kinetics inside the homochiral domain is slowed down due to a higher activation energy caused by the gain of energy due to the strong chiral discrimination, and the chiral order is preserved. Still one needs to explain the observed break of symmetry since the kinetics seems only to enhance the separation but it does not favour one of the chiral species. The break of symmetry will appear when one of the homochiral domains percolates in the fluid. In a *confined geometry* of e.g. a fluid in a bottle or a prebiotic racemic mixture in a solid chamber [9], one of the homochiral domains will dominate when, by a fluctuation, it percolates in the confined volume and encapsulates the other domains. It is a matter of *chance* which of the two enantiomers dominates and percolates by fluctuations in the system. The domain-catalytic behaviour is *selfstabilizing*, and the dominance of a percolating domain is selfstabilizing as well because its concave interface will ensure the dominance (for details see [4]).

**Figure 1.**
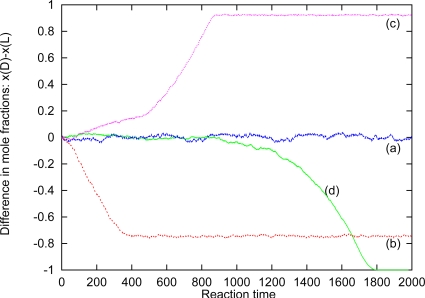
Difference in particle fraction, as a function of time in a molecular dynamics system with isomerization kinetics and for different strength of chiral discrimination, accordingly to Eq.(4): (a) | Δ_cd_*H* |= *RT*, (b) 2*RT*, (c) 3*RT* and (d) ∞.

Figure 1 shows the time evolution of chiral dominance in a fluid of particles with chiral discrimination, obtained by a realistic (molecular dynamics) computer simulation. The kinetics are implemented at time *t* = 0 and the strength of the chiral discrimination is given by the difference in the two activation energies, *E*_DL_ and *E*_DD_ = *E*_LL_. The dominance of a species is measured by the difference in molfraction, *x*_D_ – *x*_L_. For *E*_DD_ – *E*_DL_ = *RT* we do not obtain a break of symmetry even after very long times, and inspection of the (local) composition shows that the mixture remains in the racemic state. This is also to be expected since the mixing entropy is *Rln*(2). But for a stronger chiral discrimination one observes a break of symmetry and a dominance of one of the enantiomers at late times. The figure only shows four cases; but many repeated and independent simulations give an equal dominance of the D- and L-systems, within the statistical uncertainties, as one shall demand for a racemic system at the start of the simulations. The dominance appears already for a strength of 2*RT* (b in the figure), but this strength is not sufficient to kill the isomerization kinetics within the dominating domain, and the system ends in a chiral fluid, but with a small homogeneous concentration of the loosing species. (Only for an infinite strong chiral dominance of *E*_DD_ = *E*_LL_ = ∞ (d in the figure) does this system end in a pure homochiral fluid.) Two other fluid systems have been investigated [10, 11] in order to determine the sensitivity of the break of symmetry of the actual fluid systems, the conclusion being, that the isomerization kinetics which destabilizes homochirality in diluted solutions might ensure homochirality at high concentrations, provided that the chiral discrimination is sufficiently strong.

## 4. A Prebiotic Environment with Homochiral Molecules

The prebiotic environment at the origin of homochirality and of life was very different from today’s biological sphere and this fact excludes some possibilities as to where the self-assembly of prebiotic molecules and the synthesis of the templates for life could take place. After the Earth was created about 4.56 billion year ago there must have been an extensive release of gases like H_2_, N_2_, NH_3_, H_2_O, CO_2_ and CH_4_, a release which still takes place e.g. at the ”black smokers”. The energy from the Sun was only 75 % of today’s energy, and the Moon was quite close to the Earth. It is known that, most likely, there were oceans before the ”Late heavy Bombardment” about 4 billion years ago, but this bombardment created on the Earth the Hadean Ocean, named after the Greek underworld by the American geologist Preston Cloud. And for good reason, since this ocean must have been a hell for the synthesis of prebiotic template-molecules, and for life. There are several reasons why the bio-synthesis could not have taken place in or at the surface of this ocean. If the ocean was not covered by ice, there must have been huge tide waves, and with a day of only 4 hours this would result in a stirring and thereby a dilution of the concentrations. As pointed out in the introduction, a diluted aqueous solution of prebiotic chiral molecules results in a racemic composition. Furthermore, complex synthesis in a diluted solution is not possible because all steps in the consecutive biochemical reactions would then be due to (extremely) rare events. Molecular self-assembly and chemical synthesis of complex systems must take place at high concentrations of the substrates.

With these facts in mind one notices that the ”black smokers” of hydrothermal reactors in compartments [9] or in the cracks and caves beneath the smokers fulfill the conditions for an environment which can maintain homochirality, and in which self-assembly can take place. There are many experimental observations which support this hypothesis [9, 12]. The syntheses of many of the central biochemical molecules from the volatiles at the black smokers are, in general, exergonic [13, 14] at the alkaline condition. The volatiles of methane, hydrogen and carbon dioxide must have created a pool of simple biochemical molecules with Gibbs free energies favorable for a spontaneous self-assembly and synthesis of bio-systems [14, 15]. The pool of biomolecules most likely also contained the ingredients necessary for spontaneous creation of esters and ethers in the membranes [16]. Glyceraldehyde and glycolaldehyde are believed to have had a central role in the synthesis of bio-molecules [17, 18, 19]. To conclude, the confined geometry of the environment at or beneath the hydrothermal reactors fulfils the conditions for a prebiotic environment with a high concentration of the inorganic components necessary for the synthesis of simple organic molecules.

In trying to locate a molecular candidate for self-assambly to homochirality by an active kinetics, and the conditions for the break of symmetry one has several criteria to look at [20].

1. First of all a molecule should exhibit a strong chiral discrimination in favor of homochirality.
2. It should have an active isomerization kinetics at relevant temperatures and pressures.
3. It should be present in a big amounts at the origin of life.
4. If the origin of life started by obtaining homochiral domains of stereospecific molecules, one should expect these molecules to be central for the succeeding synthesis of stereospecific bio-molecules. Therefore one should expect that the molecule(s) show(s) up in bio-systems today as ”key-molecule(s)”.

In Table 1 the first criterion is only met by glyceraldehyde; but there might very well be other sugar molecules as well as amino acids or other central bio-molecules with a sufficiently strong chiral discrimination in favor of homochirality.

The second criterion points in the direction of a sugar molecule. The isomerization kinetics for such molecule is order of magnitude faster than the corresponding kinetics for amino acids, whereby the proposed mechanism can be established and maintained within relatively short times (days or years).

Also the third criterion is in the favour of sugar. Formaldehyde, CH_2_O, is the simplest carbohydrate and can be synthesized from carbon dioxide. It is produced in a reducing atmosphere containing CO_2_ and methane [21]; but more importantly it is also synthesized from volcano volatiles by reduction of CO_2_ with either methane, hydrogen or sulfide minerals. [23] The formose reaction is the spontaneous condensation of formaldehyde into sugar and was discovered already in 1861 [22]; the aldol-like mechanism is described in [24]. The condensation into sugar is catalyzed not only by amino acids [25], but also by naturally occurring aluminosilicates at hydrothermal springs and in the black smokers [26]. So the formose reaction is well known and is believed to be the source of sugars and related template molecules at the origin of life [26, 27].

In [11] and [20] it is argued that the fourth criterion also point toward glyceraldehyde as the molecule which ensured homochirality. The arguments are that D-glyceraldehyde-3-phosphate has a central role in the glycolysis and that the reduced form, Glycerol is the anchor molecule in membrane molecules. But another and very striking feature of key molecules in biology is the role of phosphate and polyphosphate despite the fact that polyphosphate is not very stable in aqueous solutions. It appears not only in D-glyceraldehyde-3-phosphate, but also in RNA and ATP-ADP, to give some well known examples. Unfortunately, there do not exists data for the chirale discrimination of e.g. D-glyceraldehyde-3-phosphate, D-ribose-1-phosphate or D-ribose-5-phosphate, and it remains to be demonstrated that a concentrated racemic solution of one of these molecules spontaneously behaves homochirally.

It is, however, not sufficient to obtain and maintain homochirality for only one of the class of constituents in biosystems, e.g. for D-glyceraldehyde or a related molecule. It is also necessary to establish the dominance of L-amino acids over the same long periode of time. There is, however, two experimental works which demonstrate a stereo-specific coupling between D-sugar and L-amino acids: L-serine is observed to make complexes with D-glyceraldehyde in aqueous solutions [28], as well as with some other L-amino acids [29]. Another example is the synthesis of tetroses from glycolaldehyde in presence of a L-dipeptide, which gives a dominance of D-tetroses [30]. If the origin and preservation of homochirality is obtained by the mechanism described in Section 3 one can understand why biosystems constitutes of D-sugar and L-amino acids and not their mirror molecules. In principle an isomerization kinetics in a racemic mixture of molecules with strong chiral discrimination will not favor one of the two chiral conformations. But with the presence of a small excess concentration of e.g. L-serine these amino molecules will perform complexes with D-glyceraldehyde molecules and act as seeds for creation of domains of D-glyceraldehyde and lead to a dominance of the D-conformation. On the other hand a dominance of the D-glyceraldehyde will enforce a dominance of L-serine.

Finally it shall be emphasized that the establishment of homochirality in a prebiotic fluid of simple chiral molecules do not resolve the fundamental problem about the origin of life and the synthesis of the template molecules for life such as RNA. For recent article about the synthesis of RNA see [31, 32].

## 5. Concluding Remarks

The molecular evolution takes place as a non-equilibrium ”self-assembling” toward higher degree of complexity. From a thermodynamical point of view this non-equilibrium self-assembling and biosynthesis is governed by the law of thermodynamics and given by the competition between entropy and energy. It is natural to assume that also the origin of chirality and the origin of life is governed by the same two driving forces. J. Monod has discussed the effect of entropy in his book ”Le hasard et la nécessité” [33] where the ”hasard”- or chance of mutations is related to the entropy. According to the prologue in the book the title is due to Demokritos who claimed that ” all what exist in the universe is the fruit of the chance and the necessity”. A thermodynamicist will, however, formulate it as ”all states of matter in the universe are obtained by decreasing the free energy either by decreasing the energy (enthalpy) or increasing the entropy”.

The law of evolution was formulated by C. Darwin as a selection by ”survival of the fittest”. The success of a mutation and a new species depends accordingly to Darwin on its ability to adapt to the environment; but this law does of course not function for a prebiotic fluid, and the law can not explain why one of of the enantiomers dominates in the evolution if there, at the origin, were a racemic mixtures of chiral pairs.

Historically there are two kind of explanations of the origin of homochirality. One is that there was a dominance already at the origin of life due to the parity-violating forces [34]. The other explanation is originally formulated by Frank [35] and explains the break of symmetry by autocatalsysis during the synthesis, either e.g. due to enantiomeric autocatalysis during the biosynthesis [31, 32, 36], or due to crystallisation [37] or other autocatalysing mechanism. But as pointed out in the introduction it is not sufficient to explain the origin of chirality, it is even more necessary to explain the preservation of homochirality over long time in a prebiotic fluid at the beginning of the evolution. The principle- or the law which ensures such a break of symmetry and the preservation of chirality is here explained as selfstabilizing chance. It is fundamentally different from the evolutionary principle of survival of the fittest in that the growth of a new species is selfstabilyzing, and the survival of the species does not depend on its ability to adapt to an ”unfriendly” environment which were there at the time of the creation and where the species must ”fight” and fit in in order to survive. The origin of chirality in a prebiotic fluid is obtained by selfstabelizing homochiral domains accordingly to this mechanism. But the selfstabelizing change acts in all the selective steps in the evolution; e.g. at the autochatalytic biosynthesis (the Frank-models). The two laws are complementary as is the two laws of thermodynamics.

The selfstabilizing effect which ensures the preservation of homochirality is an *energy* effect caused by the chiral discrimination and established by the isomerization kinetics. It is autocatalytic as in the Frank model; but it is the homochiral domains which enhances the homochirality. The chance is an *entropy* effect and plays a role in the spontaneous formation of local domains and the growth of these, and the entropy plays a role at the decisive event when a domain (in a confined geometry) encapsulates other domains of the opposite chirality. There is a qualitative difference between the Frank models for homochirality and the present model in that the Frank autocatalytic models explain the break of symmetry as taking place ”on the fly” of the biosynthesis with a complex autocatalytic kinetics of biomolecules, whereas the present explanation is that the homochirality came first and the self-assembling took place in a chiral ordered fluid.

## Figures and Tables

**Table 1.** Strength of chiral discrimination for different molecules.

Molecule	Δ_cd_*H*^⊖^/kJ mol^−1^
Glyceraldehyde(l)	− 11.7
Phenylalanine(c)	− 6.7
Isoleucine(c)	− 5.4
Lysine(c)	≈ 0.
Valine(c)	≈ 0.
Leucine(c)	3.1
Serine(c)	6.3
Proline(c)	16.6
Threonine(c)	16.9
Alanine(c)	18.9

Data taken from NIST Standard Reference Database Number 69 and references therein.

## References

[1] Bada JL (1972). Kinetics of racemization of amino acids as a function of pH. J. Am. Chem. Soc.

[2] Fedoroňko M, Königstein J (1969). Kinetics of mutual isomerization of trioses and their dehydration to methylglyoxal. Coll. Czechoslov. Chem. Commun.

[3] Pasteur ML (1850). Recherches sur les Propriétés Spécifiques des deux Acides qui composent lÁcide Racemique. Ann. Chim. Phys.

[4] Toxvaerd S (2000). Molecular dynamics simultions of isomerization kinetics in condensed fluids. Phys. Rev. Lett.

[5] Baer E Flehmig HH Refutation of alleged differences in the energy contents of optical isomers Can J Biochem 1969 47 79 83Conserning the physical chemical state (l) see footnote to Table I. 5774816 10.1139/o69-016

[6] Nagorski RW, Ricard JP (2001). Mechanistic imperatives for aldose-ketose isomerization in water: Specific, general base- and metal ion-catalyzed isomerization of glyceraldehyde with proton and hydride transfer. J. Am. Chem. Soc.

[7] Atkins P de Paula J Atkins Physical Chemistry7th Ed Oxford University Press New York, USA 2002Chapter 27.5.

[8] Toxvaerd S (2005). Homochirality in bio-organic systems and glyceraldehyde in the formose reaction. J. Biol. Phys.

[9] Russell MJ, Martin M (2004). The rocky roots of the acetyl CoA pathway. Trends. Biochem. Sci.

[10] Toxvaerd S (2004). Droplet formation in a ternary-fluid mixture: Spontaneous emulsion and micelle formation. J. Phys. Chem.

[11] Toxvaerd S (2004). Domain catalyzed chemical reactions: A molecular dynamics simulation of isomerization kinetics. J. Chem. Phys.

[12] Martin M, Russell MJ (2003). On the origins of cells : a hypothesis for the evolutionary transitions from abiotic geochemistry to chemoautotrophic prokaryotes, and from prokaryotes to nucleared cells. Philos. Trans. R. Soc. London Ser.

[13] Shock EL (1992). Chemical environments of submarine hydrothermal systems. *Origin Life Evol.*. Biosphere.

[14] Amend JP, Shock EL (2001). Energetics of overall metabolic reactions of thermophilic and hyperthermophilic archea and bacteria. FEMS Microbiol. Rev.

[15] Davis BK (1998). The forces driving molecular evolution. Prog. Biophys. Mol. Biol.

[16] Weber AL (1991). Origin of fatty acid synthesis : Thermodynamics and kinetics of reaction pathways. J. Mol. Evol.

[17] Weber AL (1987). The triose model: Glyceraldehyde as a source of energy and monomers for prebiotic condensation reactions. Origin Life.

[18] Weber AL (1992). Prebiotic sugar synthesis: Hexose and hydroxy acid synthesis from glyceraldehyde catalyzed by iron(iii) hydroxide oxide. J. Mol. Evol.

[19] Weber AL (1997). Prebiotic amino acid thioester synthesis: Thioldependent amino acid synthesis from formose substrate (formaldehyde and glycolaldehyde) and ammonia. *Origin Life Evol.*. Biosphere.

[20] Toxvaerd S (2005). Origin of homochirality in biological systems. Intern. J. Astrobiol.

[21] Schlesinger G, Miller SL (1983). Prebiotic synthesis in atmospheres containing CH_4_, CO, and CO_2_. J. Mol. Evol.

[22] Butlerow A (1861). Formation synthetique dune substance sucree. Compt. Rend. Acad. Set.

[23] Vladimirov MG, Ryzhkov YF, Alekseev VA, Bogdanovskaya VA, Otroshchenko VA, Kritsky MS (2004). Elektrochemical reduction of carbon dioxide on pyrite as a pathway for abiogenic formation of organic molecules. Origin Life Evol. Biosphere.

[24] Breslow R (1959). On the mechanism of the formose reaction. Thetrahedron Lett.

[25] Weber AL (2001). The sugar model: Catalysis by amines and amino acid products. Origin Life Evol. Biosphere.

[26] Gabel NW, Ponnamperuma C (1967). Model for origin of monosaccharides. Nature.

[27] Washington J (2000). The possible role of volcanic aquifers in prebiologic genesis of organic compounds and RNA. Origin Life Evol. Biosphere.

[28] Kock KJ, Gozzo FC, Nanita SC, Takats Z, Eberlin MN, Cooks RG (2002). Chiral transmission between amino acids: Chirally selective amino acid substitution in the serine octamer as a possible step in homochirogenesis. Angew Chem Int Ed.

[29] Takats Z, Nanita C, Cooks RG (2003). Serine octamer reactions: Indicator of prebiotic relevance. Angew. Chem. Int. Ed.

[30] Weber AL, Pizzarello S (2006). The peptide-catalyzed stereospecific synthesis of thetroses: A possible model for prebiotic molecular evolution. Proc. Natl Acad. Sci. USA.

[31] Root-Bernstein R (2007). Simultaneous origin of homochirality, the genetic code and its directionality. BioEssays.

[32] Tamura K (2008). Origin of amino acid homochirality: Relationship with the RNA world and the origin of tRNA aminoacylation. BioSystems.

[33] Monod J (1970). Le hasard et la nécessité.

[34] Szabó-Nagy A, Keszthelyi L (1999). Demonstration of the parity-violating energy difference between enantiomers. Proc. Natl. Acad. Sci. USA.

[35] Frank FC (1953). On spontaneous asymmetric catalysis. Biochim. Biophys. Acta.

[36] Sandars PGH (2003). A toy model for generation of homochirality during polymerization. Orig. Life. Evol. Biosph.

[37] Kondepudi DK, Kaufman RJ, Singh N (1990). Chiral symmetry breaking in sodium chlorate crystallization. Science.

